# The m6A Methyltransferase METTL3 Is Functionally Implicated in DLBCL Development by Regulating m6A Modification in PEDF

**DOI:** 10.3389/fgene.2020.00955

**Published:** 2020-08-27

**Authors:** Yingying Cheng, Yuanyuan Fu, Ying Wang, Jinbi Wang

**Affiliations:** ^1^Department of Hematology, The First Affiliated Hospital, College of Clinical Medicine of Henan University of Science and Technology, Luoyang, China; ^2^Department of Hematology, Changzhou Traditional Chinese Medicine Hospital, Changzhou, China

**Keywords:** METTL3, DLBCL, PEDF, *N*^6^-methyladenosine, proliferation

## Abstract

Diffuse large B-cell lymphoma (DLBCL) is the most common subtype of lymphoma, whose treatment still has a major challenge of achieving a satisfactory curative effect. The underlying mechanisms also have not been fully illustrated. *N*^6^-Methyladenosine (m6A) has been identified as the most prevalent internal modification of mRNAs present in eukaryotes, which is involved in the pathogenesis of cancers. It remains unclear how m6A mRNA methylation is functionally linked to the pathogenesis of DLBCL. In this study, we sought to explore the roles of METTL3 on DLBCL development. The results showed that m6A level for RNA methylation and the expression level of METTL3 were upregulated in DLBCL tissues and cell lines. Functionally, downregulated METTL3 expression in DLBCL cells inhibited the cell proliferation ability. Further mechanism analysis indicated that METTL3 knockdown abates the m6A methylation and total mRNA level of pigment epithelium-derived factor (PEDF). However, Wnt/β-catenin signaling was not thus activated. Overexpressed PEDF abrogates the inhibition of cell proliferation in DLBCL cells that is caused by METTL3 silence. In summary, the above-mentioned results demonstrated that the METTL3 promotes DLBCL progression by regulating the m6A level of PEDF.

## Introduction

Diffuse large B-cell lymphoma (DLBCL) is the most common subtype of lymphoma, representing 30–40% of all cases with adult non-Hodgkin lymphoma (Cheson, 2020). While approximately 10% of patients with DLBCL can be cured by the first-line treatment regimen currently used in clinics (Shimazu and Nohgawa, 2019; Morin and Scott, 2020), combined autologous hematopoietic stem cells with the present rescue chemotherapy regimen can only cure around 10% of all cases with relapsed and refractory DLBCL (Miao et al., 2019). It has become a major challenge for the present DLBCL treatment to enhance the therapeutic effects of the remaining 30% patients. A better understanding of the mechanisms underlying the formation and progression of DLBCL would contribute to the identification of the potential therapeutic targets as well as development of novel treatment regimens. In recent years, a growing number of genes involved in the formation and development of DLBCL have been identified along with the advancement of technology. It is also a research hotspot to determine how these genes exert regulatory roles in the pathogenesis of DLBCL.

*N*^6^-Methyladenosine (m6A) has been identified as the most prevalent internal modification of mRNAs present in eukaryotes (Zhuang et al., 2019). While m6A methylation is installed by a methyltransferase complex comprising methyltransferase-like 3 (METTL3), methyltransferase-like 14 (METTL14), and the associated proteins [6], this modification can be removed by alkylation repair homolog protein 5 (ALKBH5) or fat mass and obesity-associated protein (FTO), the m6A demethylases (Wei et al., 2018). In mammalian cells, coordinated regulation of the m6A methyltransferases and demethylases is crucial for maintaining this dynamic and reversible RNA modification. It has been shown that METTL3 acts in the pathogenesis of various diseases, including cancers (Zheng W. et al., 2019; Sergeeva et al., 2020; Xie et al., 2020; Yang et al., 2020). However, the role of METTL3 in DLBCL progression and the underlying mechanism have yet to be investigated.

The Wnt pathway is involved in a variety of biological and pathological processes, such as tissue homeostasis, organogenesis, stem cell regulation, and tumor development (Bernatik et al., 2020; Leibold et al., 2020). Upon activation, canonical Wnt ligands bind to the transmembrane receptor Frizzled and coreceptor LRP5/LRP6, inhibiting the degradation of cytoplasmic β-catenin. In this case, accumulated β-catenin in the cytoplasm translocates into the nucleus, where it binds to the transcription factors, regulating the expression of the target genes. The pigment epithelium-derived factor (PEDF) has been shown to act as an upstream regulator in the Wnt signaling pathway and has been implicated in a range of physiological and pathological activities (Park et al., 2011; Protiva et al., 2015; Gong et al., 2016; Ma et al., 2017). So far, it remains unclear how PEDF is functionally linked to m6A mRNA methylation in the pathogenesis of DLBCL.

In the present study, we showed an increase in the bulk m6A RNA methylation as well as METTL3 expression detected in DLBCL tissues and cell lines. Moreover, METTL3 knockdown inhibited DLBCL cell proliferation. Mechanistic studies revealed that silencing METTL3 in the DLBCL cells led to a reduction in m6A methylation in PEDF transcripts and mRNA expression of PEDF, thereby inhibiting Wnt signaling activities. Meanwhile, PEDF overexpression abolished the inhibitory effects of METTL3 knockdown on DLBCL cell proliferation. In all, these findings suggest that METTL3 facilitates DLBCL cell proliferation through regulating m6A modification in PEDF mRNAs as well as Wnt signaling activities. Thus, METTL3 may have a therapeutic potential for DLBCL.

## Materials and Methods

### Tissue Samples

A total of 36 clinical specimens comprising 18 resected DLBCL lymph glands and 18 inflammatory lymph glands used in this study were collected from the Changzhou Traditional Chinese Medicine Hospital and The First Affiliated Hospital, College of Clinical Medicine of Henan University of Science and Technology. This study obtained approval from the above-mentioned hospitals, and the sample collection was conducted in accordance with the Declaration of Helsinki. All patients enrolled between 2015 and 2019 for this study provided written informed consent. The high-throughput sequencing data of DLBCL from The Cancer Genome Atlas (TCGA)^1^ were used for validation.

### Quantitative Real-Time PCR

TRIzol reagents were used to isolate the total RNA from the sample tissues or cultured cells, and the cDNA synthesis was carried out by using a One-Step RT-PCR Kit (Thermo Fisher Scientific). An ABI Vii7 system (Applied Biosystems, Foster City, CA, United States) was employed to perform the real-time PCR. GAPDH was included as a reference control. The primer sequences for each gene were presented below: the METTL3 forward 5′-AACAGAGCAAGAAGGTCGGG-3′ and the reverse 5′-GCGAGTGCCAGGAGATAGTC-3′; the METTL14 forward 5′-CTGAAAGTGCCGACAGCATTGG-3′ and the reverse 5′-CTCTCCTTCATCCAGATACTTACG-3′; the WTAP forward 5′-CAACCTCTTTAGCCAAACAAGAA-3′ and the reverse 5′-CGACAACGTGAGTCCTTA-3′; the PEDF forward 5′-CCGTCCGAGATGAACCCTT-3′ and the reverse 5′-GCTTGTTCACGGGGACTTTG-3′; the GAPDH forward 5′-TGACTTCAACAGCGACACCCA-3′ and the reverse 5′-CACCCTGTTGCTGTAGCCAAA-3′. The comparative CT method (DDCT) was applied for calculating relative gene expression.

### Western Blotting

Western blot analysis was conducted using antibodies raised against METTL3, PEDF, β-catenin, LRP5, LRP6 (phospho S1490), lamin B1, and GAPDH (Abcam, Cambridge, MA, United States) as previously described elsewhere (Gavini and Parameshwaran, 2020). GAPDH or lamin B1 was applied for loading control.

### Cell Culture

Human DLBCL cell lines SU-DHL4, OCILy10, Farage, U2932, and HBL1 as well as human B lymphocyte GM12878 cell line were obtained from the ATCC. The cells were grown in DMEM containing 10% FBS and 1% penicillin/streptomycin under normal conditions.

### Cell Proliferation Assay

Cell proliferation was determined using the Cell Counting Kit-8 (CCK-8) (Beyotime, Shanghai, China) as described elsewhere (Lu et al., 2020). For MTT assay, transfected DLBCL cells were seeded at 1 × 10^4^ cells per well in 96-well plates and incubated for 24, 48, 72, and 96 h periods. Then, 10 μl of MTT (5 mg/ml) was applied to each well for an additional 4 h of incubation. After the supernatants were removed, 100 μl of DMSO was added to each well. The OD values were measured at 490 nm using a microplate reader.

### Lentivirus Production Transfection

The short hairpin RNA lentiviral expression plasmids targeting METTL3 and human PEDF cDNA lentivirus (LV-PEDF) were provided by Shanghai Genelily BioTech Co., Ltd. Forty-eight hours following transfection, the cells were selected with 2 μg/ml of puromycin for 2 weeks. Then, generation of cell lines with silenced expression of METTL3 was performed, and the transfection efficacy was determined by using RT-qPCR. Invitrogen Lipofectamine 3000 was used to conduct the plasmid transfection as instructed by the manufacturer.

### Flow Cytometry

Flow cytometry was conducted as described elsewhere (Klanova et al., 2019). A flow cytometry-based detection of apoptosis was performed using FITC-conjugated annexin V early apoptosis kit. Transfected DLBCL cells with si-METTL3 or negative control (NC) were subjected to an analysis on FACScan flow cytometer (BD Biosciences) and then calculated using CellQuest software from BD Biosciences.

### TOP/FOP-Flash Reporter Assay

TOP/FOP-Flash reporter assay was carried out as indicated elsewhere (Klanova et al., 2019). In brief, DLBCL cells were seeded into a 24-well plate and then transfected with the TOP/FOP-Flash plasmids (Simo Biomedical Technology, Shanghai, China). The Promega Dual Luciferase Assay kit was used to assay the luciferase activity.

### m6A Quantification

The bulk m6A of total RNA isolated from the tissue or cells was determined by using the Abcam m6ARNA Methylation Assay Kit as indicated previously (Zhu et al., 2020). Briefly, 200 ng of sample RNA (2 μl), NC (2 μl), and diluted positive control (2 μl) were then added into the designated wells with binding solution and were incubated at 37°C for 90 min. Then, the binding solution was removed. Fifty microliters of the diluted capture antibody, diluted detection antibody, and diluted enhancer solution was added to each well in order and was incubated at room temperature for 30 min each. Then, 100 μL of developer solution was added and incubated at room temperature for 10 min away from the light. The developer solution will turn blue in the presence of sufficient m6A. The absorbance was read on a microplate reader at 450 nm.

### Me-RIP Assay

The methylated m6A RNA immunoprecipitation (me-RIP) assay was carried out as indicated elsewhere (Niu et al., 2019). In brief, DLBCL cells (1 × 10^7^) were firstly lysed with RIP lysis buffer. The extracted cells and anti-m6A antibodies (3 μg in 500 μl) conjugated with magnetic beads were co-incubated at 4°C for 6 h. Following the removal of the proteins with beads, quantitative real-time PCR (qRT-PCR) was employed to detect the methylated PEDF RNA.

### RNA Stability Assay

The stability of PEDF transcripts was measured as indicated elsewhere (Zhang et al., 2020). Briefly, actinomycin D (5 mg/ml) was added to stop transcription, and samples at 0, 3, and 6 h decay were collected. ERCC RNA spike-in control (Ambion) was added to each sample before the isolation of mRNA to correct the decrease of the whole mRNA population during RNA decay. Then, qRT-PCR was employed to measure the PEDF transcripts.

### Generation of DLBCL Cell-Bearing Mice

Intraperitoneal injection of DLBCL cells labeled with fluorochrome into BALB/c nude mice (aged 4–5 weeks) was performed to generate the mouse model bearing DLBCL cells. Three weeks following the injection, a Xenogen IVIS-200 *in vivo* imaging system (Caliper Life Sciences, Hopkinton, MA, United States) was utilized to observe and analyze the bioluminescence images of intraperitoneal tumors in the mice.

### Immunofluorescent Staining (IF)

The sections were incubated with rabbit monoclonal anti-β-catenin (ab32572, 1:250) at 4°C overnight and then incubated with Alexa^®^ 488-conjugated goat anti-rabbit secondary antibody (Thermo Fisher, Waltham, MA, United States). The nuclear stain Hoechst 34580 (5 μg/ml; Molecular Probes, Thermo Fisher, Waltham, MA, United States) was added prior to final washes after the incubation of secondary antibody. Images were collected via an Olympus confocal laser scanning microscope. DAPI was used for nuclear counterstaining.

### Statistics

All data were presented as means ± SEM. Comparisons between two groups were performed by unpaired two-tailed Student’s *t*-test. ANOVA or repeated ANOVA, followed by Bonferroni *post hoc* test, was conducted for multiple comparisons using GraphPad Prism^®^ version 6.0 software. Statistically, a *p* value < 0.05 was adopted for significance.

## Results

### m6A RNA Methylation Levels and METTL3 Expression Are Increased in DLBCL

**FIGURE 1:**
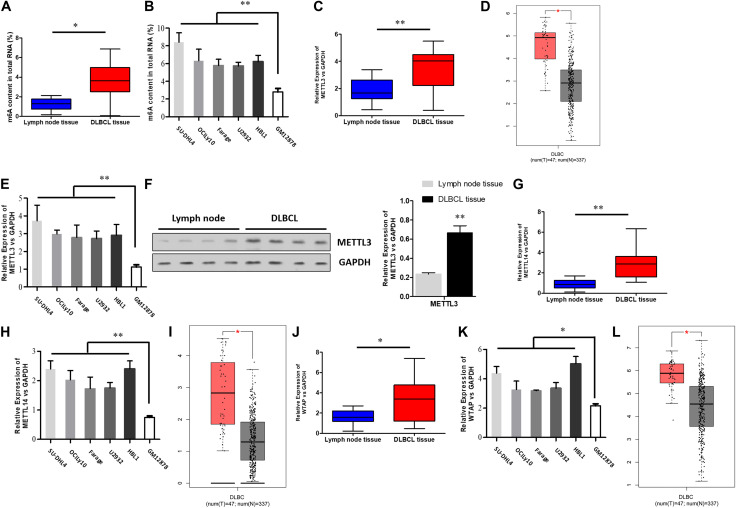
The bulk m6A RNA methylation and METTL3 expression are increased in DLBCL. **(A)** The bulk m6A RNA methylation in 18 DLBCL tissues and 18 inflammatory lymph glands. ^∗^*P* < 0.05. **(B)** The bulk m6A RNA methylation in DLBCL cell lines (SU-DHL4, OCILy10, Farage, U2932, HBL1) and Human B lymphocyte (GM12878). ^∗∗^*P* < 0.01. **(C)** mRNA expression of METTL3 in 18 DLBCL tissues and 18 inflammatory lymph glands was analyzed by qRT-PCR. ^∗∗^*P* < 0.01. **(D)** METTL3 expression in TCGA database between DLBCL tissues and normal counterparts. ^∗^*P* < 0.05. **(E)** qRT-PCR was used to analyze mRNA expression of METTL3 in DLBCL cell lines (SU-DHL4, OCILy10, Farage, U2932, HBL1) and Human B lymphocyte (GM12878). ^∗∗^*P* < 0.01. **(F)** METTL3 expression in 4 DLBCL tissues and 4 inflammatory lymph glands was analyzed by western blotting. ^∗∗^*P* < 0.01. **(G)** mRNA expression of METTL14 in 18 DLBCL tissues and 18 inflammatory lymph glands was analyzed by qRT-PCR. ^∗∗^*P* < 0.01. **(H)** qRT-PCR was used to analyze mRNA expression of METTL14 in DLBCL cell lines (SU-DHL4, OCILy10, Farage, U2932, HBL1) and Human B lymphocyte (GM12878). ^∗∗^*P* < 0.01. **(I)** METTL14 expression in TCGA database between DLBCL tissues and normal counterparts. ^∗^*P* < 0.05. **(J)** mRNA expression of WTAP in 18 DLBCL tissues and 18 inflammatory lymph glands was analyzed by qRT-PCR. ^∗^*P* < 0.05. **(K)** qRT-PCR was used to analyze mRNA expression of WTAP in DLBCL cell lines (SU-DHL4, OCILy10, Farage, U2932, HBL1) and Human B lymphocyte (GM12878). ^∗^*P* < 0.05. **(L)** WATP expression in TCGA database between DLBCL tissues and normal counterparts. ^∗^*P* < 0.05.

To determine if the m6A modification has a role in the pathogenesis of DLBCL, we first collected a total of 18 DLBCL tissues and 18 inflammatory lymph glands and analyzed the bulk m6A RNA methylation of these tissue samples. The characteristics of DLBCL patients were summarized in Supplementary Table S1. Compared with the control inflammatory lymph glands, a significant increase in the m6A levels was detected in DLBCL tissues (Figure 1A). Likewise, we found that DLBCL cell lines, including SU-DHL4, OCILy10, Farage, U2932, and HBL1, displayed a higher m6A level than the human B lymphocyte (GM12878) (Figure 1B). Next, we conducted an expression screening of the m6A methyltransferases. As shown in Figure 1C, we identified a consistent elevation in the mRNA levels of METTL3 in DLBCL tissues, based on qRT-PCR. This observation was in accordance with the data derived from the TCGA (Figure 1D). Moreover, while increased METTL3 expression in DLBCL tissues can be validated by western blotting (Figure 1F), we showed an upregulation in the mRNA levels of METTL3 in DLBCL cell lines (Figure 1E). Recent studies have shown that the m6A modification is deposited to RNAs by the m6A methyltransferase complex, a protein complex formed by the METTL3/METTL14 heterodimeric catalytic core and a regulatory subunit, Wilms’ tumor 1-associating protein (WTAP) (Park et al., 2019; Buker et al., 2020; Melstrom and Chen, 2020). Therefore, the expressions of METTL14 and WTAP were checked in DLBCL tissues and cell lines as well as data derived from TCGA (Figures 1G–L). The results showed that METTL14 and WTAP have similar expression patterns with METTL3 in DLBCL, which coordinates with the previous conclusion. Together, these data suggest that METTL3 may act as a pro-tumor gene involved in DLBCL pathogenesis through regulating m6A methylation.

### Silencing METTL3 Expression Leads to an Inhibition in DLBCL Cell Proliferation

**FIGURE 2:**
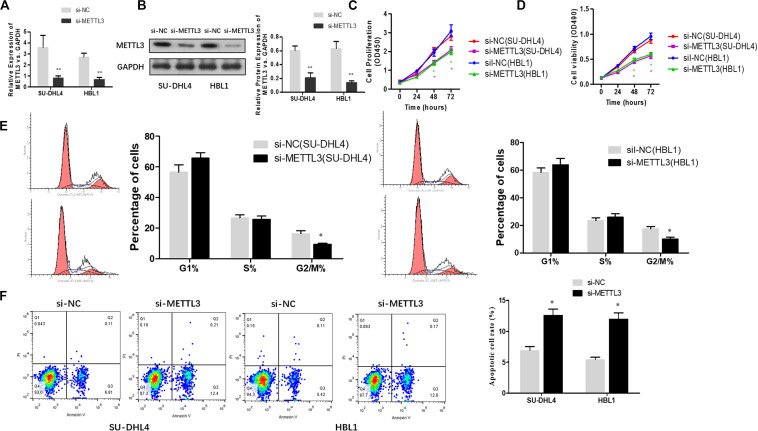
METTL3 knockdown suppresses the proliferation of DLBCL cells. qRT-PCR **(A)** and western blotting **(B)** were used to assess the efficacy of METTL3 silencing in SU-DHL4 and HBL1 cells, respectively. ^∗∗^*P* < 0.01. The viability of the DLBCL cells was determined using CCK8 **(C)** and MTT **(D)** assays. **(E)** The cell cycle distribution of the DLBCL cells was detected by flow cytometry. ^∗^*P* < 0.05. **(F)** Apoptosis in SU-DHL4 and HBL1 cells was examined by using Annexin V assay. ^∗^*P* < 0.05.

To functionally characterize METTL3 in DLBCL, we knocked down METTL3 expression using lentivirus-mediated shRNAs in DLBCL cell lines (SU-DHL4 and HBL1) and examined whether reduced expression of METTL3 affects cell proliferation. Prior to cell proliferation assays, we validated the efficacy of METTL3 expression silencing in SU-DHL4 and HBL1 cells using qRT-PCR and western blotting (Figures 2A,B). To assess the proliferative ability of the DLBCL cells with reduced expression of METTL3, we first performed CCK-8 (Figure 2C) and MTT (Figure 2D) assays. As depicted in Figures 2C,D, silencing METTL3 expression resulted in an inhibition in the proliferation of SU-DHL4 and HBL1 cells. We next determined the cell cycle status of DLBCL cells with the silenced expression of METTL3 based on flow cytometry. The analysis revealed that the proportion of G2/M-phase cells was decreased in METTL3-silenced cells in comparison with that in the control cells (Figure 2E). Meanwhile, the annexin V assay showed a higher rate of apoptotic cells in DLBCL cell lines with reduced expression of METTL3 compared with the control ones (Figure 2F). All these observations indicated that METTL3 knockdown caused an inhibition in DLBCL cell proliferation *in vitro*.

### METTL3 Knockdown Inhibits PEDF Expression and m6A Methylation in PEDF mRNA

**FIGURE 3:**
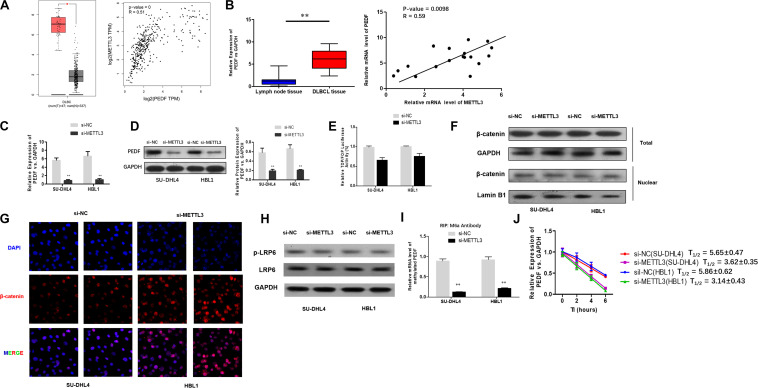
METTL3 knockdown down-regulates PEDF expression and m6A methylation in PEDF mRNAs, as well as Wnt signaling activities. **(A)** PEDF expression and correlation of the expression of METTL3 with PEDF expression in TCGA database between DLBCL tissues and normal counterparts. ^∗^*P* < 0.05. **(B)** mRNA expression of PEDF in 18 DLBCL tissues and 18 inflammatory lymph gland specimens was determined using qRT-PCR. ^∗∗^*P* < 0.01. A positive correlation between mRNA expression of METTL3 and PEDF was detected by linear regression analysis. qRT-PCR **(C)** and western blotting **(D)** were used to analyze mRNA and protein expression of PEDF in SU-DHL4 and HBL1 cells with silenced expression of METTL3, respectively. ^∗∗^*P* < 0.01. TOP/FOP-Flash reporter **(E)** was employed to determine Wnt signaling activity in SU-DHL4 and HBL1 cells with silenced expression of METTL3. **(F)** Western blotting assay of total and nuclear β-catenin proteins in DLBCL cells with silenced expression of METTL3. GAPDH and Lamin B1 were used as internal control and endogenous control of cell nuclear fraction, respectively. **(G)** Accumulation of β-catenin in the nucleus of the DLBCL cells with silenced expression of METTL3 according to confocal microscope images. **(H)** Western blotting assay of total and phosphorylated LRP6 proteins in DLBCL cells with silenced expression of METTL3. GAPDH was used as internal control. Me-RIP **(I)** assay was conducted to determine m6A methylation in PEDF transcripts in SU-DHL4 and HBL1 cells with silenced expression of METTL3. ^∗∗^*P* < 0.01. **(J)** The half-life (T1/2) of PEDF mRNAs in SU-DHL4 and HBL1 cells transfected with Lv-shMETTL3 or Lv-NC (the control lentivirus).

To investigate the mechanisms underlying the role of METTL3 silencing in inhibition of DLBCL cell proliferation, we examined if PEDF, the upstream Wnt pathway component, plays a role in METTL3-mediated effects on DLBCL. TCGA database search revealed that PEDF was overexpressed in DLBCL tissues, and further linear regression analysis identified a positive correlation between increased expression of PEDF and METTL3 expression in DLBCL tumor tissues and whole blood (Figure 3A). Similarly, our experiments showed that while mRNA expression of PEDF was markedly increased in DLBCL tissues, a positive correlation between upregulated PEDF expression and METTL3 levels was detected in those tissue samples (Figure 3B). We next sought to determine if METTL3 functions in regulating PEDF expression in DLBCL cells. As shown in Figures 3C,D, silencing METTL3 led to a reduction in both mRNA and protein expression of PEDF in SU-DHL4 and HBL1 cells. However, we found that transcriptional activity of TOP/FOP was not significantly impacted in SU-DHL4 and HBL1 cells with silenced expression of METTL3 (Figure 3E). Silencing METTL3 in SU-DHL4 and HBL1 cells also could not obviously modulate the expression of β-catenin in the nucleus and phosphorylation of LRP6 (Figures 3F–H). METTL3 silencing caused a decrease in m6A methylation in PEDF mRNAs in the DLBCL cells (Figure 3I). To determine if decreased m6A methylation affects the mRNA stability of PEDF in the cells, we carried out an RNA stability assay. The assay revealed that the half-life of PEDF gene transcripts was shortened in SU-DHL4 and HBL1 cells with silenced expression of METTL3 (Figure 3J), suggesting that decreased expression of PEDF in the DLBCL cells with METTL3 silencing can be at least partially attributed to reduced mRNA stability of PEDF linked to altered m6A methylation level. Thus, we reasoned that silencing METTL3 in DLBCL cells downregulates PEDF expression through acting on the mRNA methylation (m6A).

### PEDF Overexpression Abolishes the Inhibitory Effects of METTL3 Knockdown on DLBCL Cell Proliferation

**FIGURE 4:**
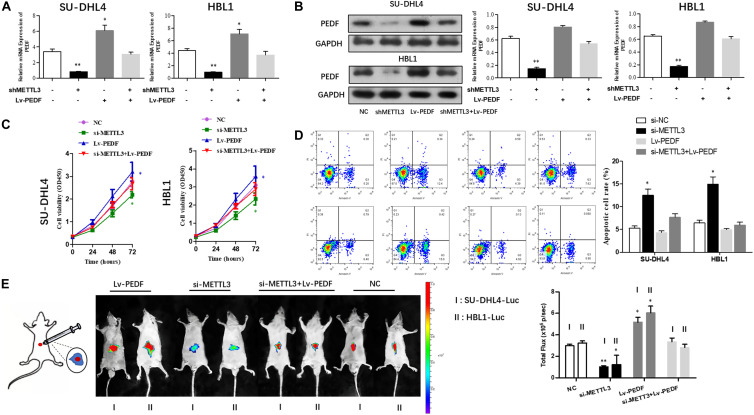
The overexpression of PEDF reverses the inhibitory effects of METTL3 knockdown on DLBCL cell proliferation. PEDF expression at mRNA **(A)** and protein levels **(B)** was determined using qRT-PCR and western blot analysis, respectively. CCK8 **(C)** and Annexin V assays **(D)** were employed to detect the viability and apoptotic rate of SU-DHL4 and HBL1 cells, respectively. **(E)** The proliferative activity of SU-DHL4 and HBL1 cells in the mouse model was assessed. Bioluminescence images of the mice bearing SU-DHL4-luc and HBL1-luc cells as well as relative luminescence intensities were illustrated. All experiments above were conducted after transfection with Lv-NC, Lv-shMETTL3, Lv-PEDF and Lv-shMETTL3 + Lv-PEDF. ^∗^*P* < 0.05, ^∗∗^*P* < 0.01 vs. the control groups.

Next, we sought to investigate if PEDF mediates the inhibitory effects of METTL3 knockdown on DLBCL cell proliferation. For this purpose, SU-DHL4 and HBL1 cells were transfected with NC, METTL3 shRNA plasmid, LV-PEDF, and shMETTL3 + LV-PEDF, respectively. As shown in Figures 4A,B, PEDF expression in the transfected cells was validated using qRT-PCR and western blotting. We then analyzed the effect of PEDF overexpression on METTL3 knockdown-mediated regulation of DLBCL cell proliferation. The cell proliferation (Figure 4C) and apoptotic assays (Figure 4D) revealed that PEDF overexpression markedly relieved the inhibitory effects of METTL3 silencing on DLBCL cell proliferation. To further test the role of PEDF *in vivo*, DLBCL cell-bearing mice were generated by intraperitoneal injection of SU-DHL4 and HBL1 cells stably expressing luciferase as well as indicated genes (SU-DHL4-Luc and HBL1-Luc) into the nude mice. Three weeks after the injection, an *in vivo* imaging system was employed to examine the mouse models. As depicted in Figure 4E, the mice bearing DLBCL cells with silenced expression of METTL3 displayed remarkably lower luminescence intensities than those in the control group, suggesting a METTL3 knockdown-induced inhibition in the tumor progression *in vivo*. Notably, we observed that the mice harboring PEDF-overexpressed DLBCL cells exhibited a comparable luminescence intensity with those in the control group. Collectively, these findings indicated that PEDF overexpression abolished the inhibitory effects of METTL3 knockdown on DLBCL cell activities *in vivo*.

## Discussion

Here, we provided the first demonstration that METTL3 and m6A RNA modifications are functionally implicated in DLBCL development. The present study showed that the bulk m6A RNA methylation and METTL3 expression were significantly increased in the tissues and cell lines of DLBCL.

*N*^6^-Methyladenosine methylation is considered one of the most prevalent chemical modifications in mRNAs and has been shown to be critically involved in cancer development. This study identified an increased expression of METTL3 in DLBCL tissues and cells. Furthermore, silencing METTL3 led to an inhibition in DLBCL cell proliferation both *in vitro* and *in vivo*, suggesting that METTL3 may act as a pro-tumor gene involved in the pathogenesis of DLBCL.

Recent studies showed that aberrant expression of METTL3 is associated with various cancers. TCGA data-based analysis has identified acute myeloid leukemia, DLBCL, and prostatic cancer as the top three malignancies with the most expression abundances of METTL3 among common tumors. These data suggest that highly expressed METTL3 may be functionally linked to the formation and progression of certain cancers. A subsequent study revealed that while METTL3 maintains leukemic cell growth via the sp1/c-MYC pathway, depletion of METTL3 leads to a cell cycle arrest and differentiation of the leukemic cells (Barbieri et al., 2017). In lung cancers, METTL3 was found to selectively promote the translation of mRNAs containing the m6A peaks around the stop codons, such as mRNA transcripts of the oncogenes EGFR and DNMT3A, and to increase the expression of the oncoproteins, thereby facilitating the proliferation and invasion of the tumor cells (Lin et al., 2016). Moreover, this study demonstrated that METTL3 recruits the initiator factor elF3 to the translational initiation complex for facilitating the translational efficacy, while the cytoplasmic METTL3 can be independent of its own or other methyltransferase activities and the N-terminus of METTL3 is capable of directly upregulating the translation of its target genes. Besides, reduced expression of METTL3 caused a marked inhibition in the proliferation and invasion of lung cancer cells, as well as an increased apoptosis. In the meantime, studies on leukemia indicated that m6A modification promotes the translation of oncogenes c-MYC, Bcl-2, and PTEN. In this case, depletion of METTL3 increased the phosphorylation of protein kinase B (AKT) and induced the differentiation and apoptosis of the leukemic cells, eliciting a delay in the progression of leukemia (Vu et al., 2017). Overall, these data support the notion that METTL3-catalyzed m6A methylation can affect the activities of certain tumor-specific mRNAs, eliciting a change in the expression of the oncoproteins and biological behaviors of the tumor cells and facilitating the cancer development. However, it is not the case that METTL3 is highly expressed in all tumors. Instead, a low expression level of METTL3 was detected in certain tumors in which upregulated expression of METTL3 could effectively inhibit the tumor development. It has been reported that kidney cancer tissues display a low expression of METTL3 that not only promotes the proliferation, growth, and colony formation of the cancer cells via the PI3K/Akt/mTOR pathway but also activates the EMT to facilitate the migration and invasion of the cells (Li et al., 2017). Notably, upregulating the expression of METTL3 also may significantly suppress the growth of solid tumors (Wang et al., 2017; Hu et al., 2020). Meanwhile, follow-up studies on prognosis showed that the patients with a high level of METTL3 expression exhibit a longer survival period.

It has been reported that aberrantly activated Wnt signaling is critically involved in DLBCL progression (Koch et al., 2014; Bognar et al., 2016). To explore the mechanism underlying the role of METTL3 in DLBCL, we analyzed the regulatory effects of METTL3 on Wnt signaling via m6A methylation in PEDF mRNA transcripts. However, we did not observe that METTL3 knockdown resulted in a corresponding regulation on Wnt signaling activities. As PEDF was usually regarded as a canonical Wnt signaling inhibitor in previous studies (Park et al., 2011; Protiva et al., 2015; Gong et al., 2016; Ma et al., 2017), this result strongly suggests that there might be a key back regulator of Wnt signaling synchronously enhanced that balances the effect of PEDF. It also indicated that canonical Wnt signaling may not be the primary pathway that affected METTL3 modulation in DLBCL cells. The back regulator and signaling involved need to be further identified through high-throughput sequencing. PEDF was widely considered as a tumor suppressor in solid tumors as it exhibits antiangiogenic and antimetastatic activities (Baxter-Holland and Dass, 2018; Nardi et al., 2018; Ansari et al., 2019; Honrubia-Gomez et al., 2019; Huang et al., 2019). Its role in hematologic malignancies such as leukemia, lymphoma, and multiple myeloma remains unclear. DLBCL cells are a sort of suspension cultured cells that exhibit different biological characteristics compared with conventional anchorage-dependent cells from solid tumors. They are freely transferred in human blood circulation in which the antiangiogenic or antimetastatic activities possessed by PEDF may not efficiently function. Therefore, PEDF presented a distinctive pro-tumor role in DLBCL in this study. Genes’ function may differ in various cancers. Erbin was reported to associate with the stage and progression in colorectal cancer (Yao et al., 2015), thereby exhibiting the characteristics of oncogenes. In contrast, Zheng Z. et al. (2019) found that depletion of Erbin in acute myeloid leukemia cells could enhance the cell proliferation and block the cell differentiation, which suggests that Erbin may exert carcinostasis in acute myeloid leukemia. The findings in this study led us to propose that increased m6A level in PEDF mRNAs may underlie METTL3-mediated regulation of DLBCL cell proliferation. However, the regulatory role of the METTL3/PEDF axis in DLBCL development needs to be further investigated.

In sum, this study presented evidence that the m6A methyltransferase METTL3 acts in DLBCL cell proliferation by regulating m6A modification in PEDF mRNAs. The METTL3/PEDF axis may have a therapeutic potential for DLBCL.

## Data Availability Statement

The raw data supporting the conclusions of this article will be made available by the authors, without undue reservation.

## Ethics Statement

The studies involving human participants were reviewed and approved by the Changzhou Traditional Chinese Medicine Hospital and The First Affiliated Hospital, College of Clinical Medicine of Henan University of Science and Technology. The patients/participants provided their written informed consent to participate in this study. The animal study was reviewed and approved by Changzhou Traditional Chinese Medicine Hospital and The First Affiliated Hospital, College of Clinical Medicine of Henan University of Science and Technology.

## Author Contributions

YC wrote the manuscript. YF prepared the figures. JW and YW edited the manuscript. All authors contributed to the article and approved the submitted version.

## Conflict of Interest

The authors declare that the research was conducted in the absence of any commercial or financial relationships that could be construed as a potential conflict of interest.

## Footnotes

1 http://gepia.cancer-pku.cn/detail.php
