# Supplementary material for: The m6A Methyltransferase METTL3 Is Functionally Implicated in DLBCL Development by Regulating m6A Modification in PEDF
Source: Front Genet. 2020 Aug 27;11:955. doi: 10.3389/fgene.2020.00955 (PMC7481464; doi:10.3389/fgene.2020.00955)
Supplement: Supplementary file 1 [file Table_1.DOCX]

**Supplementary Table 1 Summary of DLBCL patient characteristics**

| Patients/ parameter | Age | Gender | Ann Arbor stage | ECOG PS | LDH level | Extranodal involvement | B symptoms | GC phenotype | METTL3 expression vs GAPDH |
| --- | --- | --- | --- | --- | --- | --- | --- | --- | --- |
| 1 | 52 | M | II | 0 | Normal | <2 | No | Non-GC  type | 0.387 |
| 2 | 46 | M | III | 0 | Elevated | <2 | No | Non-GC  type | 4.074 |
| 3 | 42 | F | III | 0 | Normal | <2 | No | GC type | 5.277 |
| 4 | 70 | F | II | 1 | Elevated | <2 | Yes | Non-GC  type | 0.617 |
| 5 | 62 | F | III | 0 | Elevated | <2 | No | GC type | 4.007 |
| 6 | 48 | M | III | 0 | Elevated | ≥2 | Yes | Non-GC  type | 5.338 |
| 7 | 50 | F | II | 0 | Normal | <2 | No | Non-GC  type | 4.401 |
| 8 | 55 | F | III | 1 | Elevated | <2 | Yes | GC type | 4.373 |
| 9 | 62 | M | II | 0 | Normal | ≥2 | No | GC type | 3.280 |
| 10 | 65 | M | II | 0 | Normal | <2 | Yes | Non-GC  type | 2.593 |
| 11 | 58 | M | III | 1 | Elevated | <2 | No | GC type | 2.461 |
| 12 | 48 | F | II | 0 | Elevated | <2 | No | Non-GC  type | 5.486 |
| 13 | 53 | M | III | 0 | Normal | <2 | Yes | Non-GC  type | 4.821 |
| 14 | 60 | M | III | 0 | Elevated | <2 | No | GC type | 1.138 |
| 15 | 57 | M | II | 1 | Normal | <2 | Yes | Non-GC  type | 3.285 |
| 16 | 66 | F | III | 1 | Elevated | <2 | Yes | Non-GC  type | 4.276 |
| 17 | 46 | M | II | 0 | Elevated | <2 | Yes | GC type | 4.070 |
| 18 | 47 | M | III | 1 | Elevated | ≥2 | No | Non-GC  type | 1.533 |
